# East-West cultural differences in encoding objects in imagined social contexts

**DOI:** 10.1371/journal.pone.0207515

**Published:** 2018-11-20

**Authors:** Lixia Yang, Juan Li, Andrea Wilkinson, Julia Spaniol, Lynn Hasher

**Affiliations:** 1 Department of Psychology, Ryerson University, Toronto, Ontario, Canada; 2 Center on Aging Psychology, CAS Key Laboratory of Mental Health, Institute of Psychology, Beijing, China; 3 Mechanical and Industrial Engineering Department, University of Toronto, Toronto, Ontario, Canada; 4 Department of Psychology, University of Toronto, Toronto, Ontario, Canada; 5 Rotman Research Institute, Baycrest Health Sciences, University of Toronto, Toronto, Ontario, Canada; Victoria Univ Wellington, NEW ZEALAND

## Abstract

It has been shown in literature that East Asians are more inclined to process context information than individuals in Western cultures. Using a context memory task that requires studying object images in social contexts (i.e., rating objects in an imagined social or experiential scenario), our recent study revealed an age-invariant advantage for Chinese young and older participants compared to their Canadian counterparts in memory for encoding contexts. To examine whether this cultural difference also occurred during encoding, this follow-up report analyzed encoding performance and its relationship to subsequent memory based on the same data from the same task of the same sample. The results revealed that at encoding, Chinese participants provided higher ratings of objects, took longer to rate, and reported more vivid imagery of encoding contexts relative to their Canadian counterparts. Furthermore, only Chinese participants rated objects with recognized context at retrieval higher and slower relative to those with misrecognized context. For Chinese participants, primarily older adults, slower ratings were only related to better context memory but not item memory. Importantly, Chinese participants' context memory advantage disappeared after controlling for encoding differences. Taken together, these results suggest that Chinese participants' memory advantage for social contexts may have its origin in the construction of elaborative and meaningful object-context associations at encoding.

## Introduction

Accumulated research suggests East-West cultural differences in processing contextual information. Specifically, East Asians were more inclined to process context information than Westerners [1–4]. For example, Masuda and Nisbett (2001) found that Japanese participants remembered more background information, and their memory for objects was more impacted by the change or removal of the background, relative to American participants [3]. In an eye-tracking study, Chua and colleagues (2005) revealed that American participants fixated more on the focal objects, whereas Chinese participants had more eye movements to the background of a scene [1]. Similarly, East Asians attended to a broader perceptual field that likely integrated targets and background in comparison to their Western counterparts [5–7].

Given their inclination to process contextual information in most previous studies [1–4], East Asians may be differentially more engaged in processing context when required to make meaningful item-context associations, which may in turn benefit their memory for contextual information. Using an intentional context memory paradigm, our previous work examined cultural differences and age-related declines in memory for objects and their associated imagined social or experiential encoding contexts [8]. Aging-associated decline in memory for sources or contexts has been consistently documented in Western individuals [9–10]. Little is known about whether this decline might be diminished in East Asians given their holistic context-integrated information processing style. In our previous work, context was defined as an imagined social or experiential scenario in which stimuli were presented to participants for their evaluations. Specifically, participants studied images of objects by rating them in one of two imagined social scenarios (i.e., contexts) versus a culturally neutral experiential control context. The social contexts involved rating objects in terms of how meaningful/useful each object was for living independently (i.e., INDEPENDENT) in one block or getting along with others (i.e., RELATIONAL) in a new city in the other block. The experiential control context involved rating objects on their typicality in daily life (i.e., DAILY LIFE). In all these conditions, participants need to form socially and/or personally meaningful object-context associations (e.g., the uses of the object in a specific social scenario or the frequency of the object experienced in participant’ personal daily life). Memory for the objects and their associated encoding contexts (i.e., INDEPENDENT/RELATIONAL or DAILY LIFE) was subsequently tested in a context recognition task. The results suggested that the age-related declines in episodic memory equally applied to East Asians. Additionally, the results showed an age-invariant memory advantage for the encoding contexts favoring Chinese over Canadian participants [8]. However, it remains unclear whether the age-invariant cultural effects emerged during study/encoding phase.

Different from Yang et al. [8] which exclusively focused on cultural effects on memory retrieval performance, the current report analyzed the cultural differences in encoding performance and encoding-recognition relationship based on the same sample. These encoding results have never been reported in Yang et al. [8] or elsewhere. Encoding performance was indexed by ratings provided to objects in the corresponding contexts, rating response time (RT), and self-reported vividness of imagery formed for each encoding context. The encoding-recognition relationship was assessed by comparing rating performance for items as a function of their context recognition status (i.e., whether or not a context was subsequently correctly recognized), calculating encoding-memory correlations, and conducting covariance analysis on context memory controlling for encoding differences.

The current paper addresses three research questions: 1) Do Chinese and Canadian participants differ in rating objects in imagined social or experiential contexts at encoding? 2) Are the cultural differences in encoding performance seen in young adults preserved in older adults? 3) Do cultural differences in encoding, if any, relate to the cultural effects on recognition of encoding contexts? Considering that relative to Westerners, East Asians are more sensitive to and affected by context [3, 11], we predicted that Chinese participants would invest more time and generate more vivid object-context associations. Specifically, we hypothesized that: 1) Chinese participants would provide higher ratings, take longer to rate, and form more vivid encoding context imagery relative to Canadian participants; 2) the cultural difference in encoding performance would be preserved in older adults; 3) more engaged rating performance (i.e., higher rating and longer rating RT) at encoding would be related to better context memory at retrieval. These hypotheses were largely supported by the results.

## Method

The study was approved by Ryerson University Research Ethics Board (REB 2010–118) and the Ethics Board of the Institute of Psychology, Chinese Academy of Sciences. Informed written consent was obtained from all participants before their participation.

### Participants

The final sample included 71 Canadians of European descent living in Toronto (35 young, aged *M* = 20.77, *SD* = 2.95; and 36 older, aged *M* = 68.86, *SD* = 6.03) and 72 native Chinese living in Beijing (36 young, aged *M* = 20.69, *SD* = 1.56; and 36 older, aged *M* = 67.72, *SD* = 4.47). Young Canadians were recruited from an undergraduate participant pool and tested at the Cognitive Aging Laboratory at Ryerson University in Toronto whereas young Chinese were undergraduate students recruited through University campus posters and tested at the Institute of Psychology, Chinese Academy of Sciences, in Beijing. Older Canadians and Chinese were recruited through an internal participant pool and/or through posters from local communities in Toronto and Beijing respectively. One Canadian participant was excluded because of a false alarm rate of 100%, reflecting a possible misunderstanding or confusion of response keys during recognition. Table 1 displays sample characteristics.

**Table 1:** Sample characteristics.

	Canadian		Chinese	
	Young (*n* = 35)	Older (*n* = 36)	Young (*n* = 36)	Older (*n* = 36)
**Age**	20.77 (2.95)	68.86 (6.03)	20.69 (1.56)	67.72 (4.47)
**Gender (F/M)**	27/8	14/22	19/17	20/16
**Education**^**a**^	14.01 (2.23)	16.28 (3.33)	14.06 (1.17)	14.47 (2.66)
**Health**^**b**^	7.81 (1.11)	8.41 (1.35)	7.82 (1.22)	7.58 (1.27)
**Independence**^**c**^	4.96 (0.66)	5.34 (0.72)	4.49 (0.57)	4.99 (0.75)
**Interdependence**^**c**^	4.72 (0.54)	4.53 (0.68)	5.01 (0.59)	5.37 (0.58)
**CES-D**	14.31 (9.76)	8.28 (8.02)	14.36 (7.15)	5.17 (5.43)
**MMSE**	N/A	28.86 (1.17)	N/A	28.86 (1.10)

Each cell provides mean score, with standard deviation in parenthesis. F = Female; M = Male; CES-D = the Center for Epidemiological Studies Depression Scale; MMSE = the Mini-Mental State Examination.

^a^Education was measured in years of formal education

^b^Health was measured by self-report ratings based on a 1–10 Likert-Type scale.

^c^Independence and interdependence were measured with the SCS.

Overall, participants from both cultures showed satisfactory proficiency in their native language, as all Canadian participants scored above 20 on the Shipley vocabulary test [12] and all Chinese participants scored above 41 on the verbal intelligence measure of the Wechsler’s Adult Intelligence Scale (WAIS) [13]. The two cultural samples did not differ in gender distribution (*χ*^2^ = .19, *p* = .74). All older adults scored above 26 on the Mini-Mental State Examination (MMSE) [14], suggesting no dementia-related cognitive impairment. To assess potential covariates and/or verify the cultural orientation profiles of the two cultural groups, we administered a set of tests, including a background questionnaire, the Center for Epidemiological Studies Depression Scale (CES-D) [15], and the Self-Construal Scale (SCS) [16]. Older adults were lower in depression and higher in SCS independence score. For older but not young adults, Canadians reported more years of formal education and higher health ratings than their Chinese counterparts. As expected for their specific cultural norms, Canadians scored higher on independence, but lower on interdependence self-construal scores of the SCS. For details of the analyses on sample characteristics, please refer to our previous publication [8].

### Stimuli and procedure

The stimuli included 180 line-drawing pictures of common objects [17]. Based on cross-cultural norms [18], all chosen images included easily recognizable familiar objects with a high level of name or concept agreement across the two cultures. The pictures were divided into six sets of 30 pictures, counterbalanced so that each set was assigned equally often to each of the two context memory blocks. Within each block, each of the three sets was encoded equally often in the two encoding contexts or served as the new set at recognition.

Each participant completed two blocks of context memory task, called RELATIONAL and INDEPENDENT. Each block contained 60 serially presented object pictures, each with a cue word presented above. In the RELATIONAL block (i.e., R block), half of the objects were cued with “RELATIONAL” (“关系” for Chinese) to which participants were asked to imagine that they were moving to a new city and to rate how meaningful each object would be “for you to get along with others and to be liked and accepted by others” in the new city (R context) and the other half were cued with “DAILY LIFE” (“日常生活” for Chinese) to which participants rated how typical the object was experienced in daily life (D context). In the INDEPENDENT block (i.e., I block), half of objects were cued with “INDEPENDENT” (“独立” for Chinese) to which they rated how meaningful each object would be “for you to live independently” in the new city (I context) and the other half were cued with the DAILY LIFE” (“日常生活” for Chinese) for which they rated the typicality of the objects experienced in everyday life (D context). The order of the two blocks was counterbalanced, so that half of the participants completed the I block first, followed by the R block, and the other half of participants completed the two blocks in reversed order (the R block followed by the I block). They responded by pressing the corresponding number keys to rate meaningfulness/typicality based on a scale of 1 (“least meaningful/typical”) to 5 (“most meaningful/typical”). The pictures were presented in a pseudo-randomized order, with no more than three trials of the same cues in a row. Each trial started with a centered fixation cross (1000 ms), replaced by a stimulus that was terminated by a response, then followed by a 1000-ms blank screen inter-stimulus interval (ISI). Four practice trials (two I/R and two D context) were given at the beginning of each block.

Following the encoding phase of each block, a recognition task was given in which participants viewed 90 pictures (60 studied and 30 non-studied new pictures). Each picture had to be identified as an old studied in the I or R context (by pressing the "I”/“R" key) or in the D context (by pressing the “D” key), or a new one (by pressing the “N” key). The key-response mapping and response hand (left or right) were counterbalanced across participants [8]. Participants were asked to rest their response fingers on the corresponding keys and respond as quickly and accurately as possible. Pseudo-randomized sequencing was adopted such that no more than two expected responses of the same type occurred in a row. Each recognition trial started with a centered fixation cross (1000 ms), followed by a picture that would be terminated by a response (5000 ms maximum), and then replaced by a blank screen ISI (1000 ms). The recognition within each block started with six practice trials (two I/R, two D, and two new).

Following the two memory blocks, participants self-reported vividness of the imagery formed during encoding for each context (i.e., I or R, and D) by rating them based on a scale of 1 (“very slightly or not at all vivid”) to 5 (“extremely vivid”). Finally, participants completed a battery of paper-and-pencil tests in the following order: SCS, vocabulary test (i.e., Shipley for Canadians and the WAIS vocabulary subscale for Chinese), CES-D, MMSE, and a background questionnaire. For all of the tests/tasks that do not have corresponding standardized Chinese versions, the instructions were translated and back-translated by two researchers who are fluent in both English and Mandarin. Any discrepancies were resolved through a follow-up discussion.

## Results

The data were analyzed with IBM SPSS Statistics 24. The alpha level for significance was set at *p* < .05, with marginal significant effects at *p* < .10. Greenhouse-Geisser correction was used to adjust the degrees of freedom when Mauchly’s test was significant, suggesting a violation of the sphericity assumption. Shapiro-Wilk's tests confirmed that ratings across all item types did not deviate significantly from the normal distribution (*p*s > .23). However, the normality assumption was violated for rating RTs (*p*s < .001). RT data were thus log transformed before they were submitted to the analysis of variance (ANOVA). Bonferroni correction of the alpha level was applied for all post-hoc multiple comparisons. The results are reported in two sections: 1) encoding performance (i.e., rating, rating RT, and self-reported vividness of encoding context imagery); 2) encoding-memory relationship (i.e., rating performance as a function of items’ context recognition status, rating-memory correlations, and covariance analysis on context memory controlling for encoding differences).

### Encoding performance

Ratings and log transformed rating RTs were each submitted to a 2 (culture: Canadian vs. Chinese) × 2 (age: Young vs. Old) × 4 (item type: I, R, DI, and DR) mixed model ANOVA (General Linear Model, GLM in SPSS), with item type as the only within-subjects variable. DI stands for D items in the I block, whereas DR stands for D items in the R block. As an index of context encoding quality, the self-reported vividness of mental imagery formed for each of the I, R, and D contexts during encoding were submitted to a 2 (culture) × 2 (age) × 3 (context: I, R, and D) mixed model ANOVA, with context as the only within-subjects variable.

#### Rating

**Fig 1:**
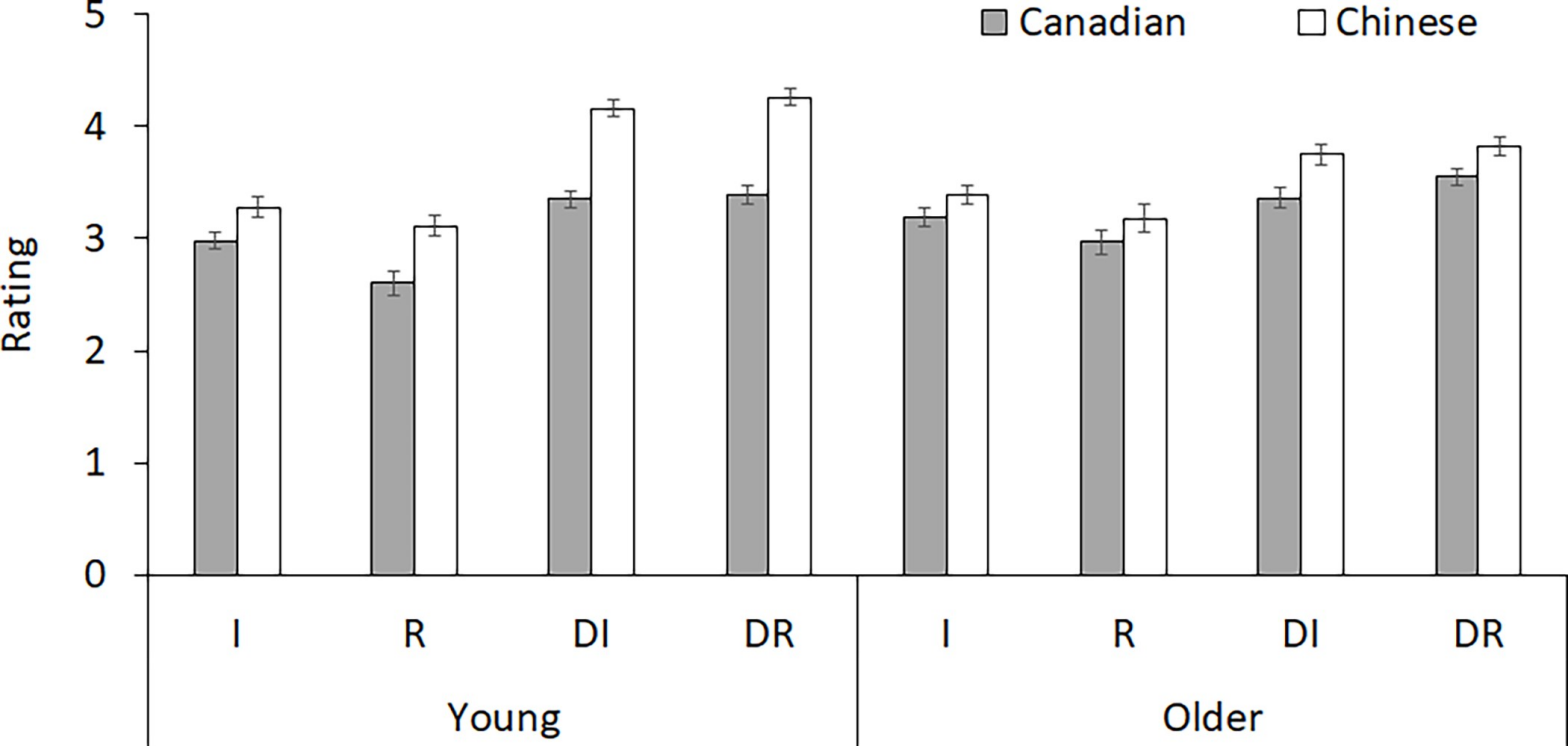
Ratings at encoding for each age and culture group across item types. R = Relational items; I = Independent items; DR = Daily life items in the “Relational” block, DI = Daily life items in the “Independent” block.

The ANOVA on ratings at encoding (see Fig 1) revealed a main effect of culture, *F*(1,139) = 47.72, *MSE* = 0.59, *p* < .001, η^2^ = 0.26, with higher ratings by Chinese (*M* = 3.62, *SD* = 0.39) than by Canadian participants (*M* = 3.18, *SD* = 0.39). This was qualified by a culture by age interaction, *F*(1,139) = 7.65, *MSE* = 0.59, *p* < .01, η^2^ = 0.05. Follow-up analyses showed that Canadian older adults provided higher ratings relative to Canadian young adults (*p* < .05), whereas Chinese tended to show the opposite pattern, with higher ratings by young than older adults (*p* = .07). Nevertheless, both age groups showed a significant cultural effect (*p*s < .01). The main effect of item type was also significant, *F*(2.38,330.58) = 105.28, *MSE* = 0.19, *p* < .001, η^2^ = 0.43. Pairwise comparisons with Bonferroni correction showed that ratings were higher for DR (*M* = 3.76, *SD* = 0.59) than DI items (*M* = 3.66, *SD* = 0.58), which were rated higher then I items (*M* = 3.21, *SD* = 0.51), and I items were in turn rated higher than R items (*M* = 2.97, *SD* = 0.70), *p*s < .05. This item type effect was qualified by an interaction with culture, *F*(2.38,330.58) = 5.45, *MSE* = 0.24, *p* < .01, η^2^ = 0.04; and an interaction with age, *F*(2.38,330.58) = 8.65, *MSE* = 0.24, *p* < .001, η^2^ = 0.06. Follow-up analyses suggested that although both culture or age groups showed significant item type effect (*p*s < .001), the effect size was larger for Chinese (*F* = 66.82, η^2^ = 0.49) than for Canadian participants (*F* = 35.32, η^2^ = 0.34), and larger for young (*F* = 86.28, η^2^ = 0.55) than for older adults (*F* = 26.07, η^2^ = 0.27). No other effects were significant (*p*s > .10).

#### Rating RT

**Fig 2:**
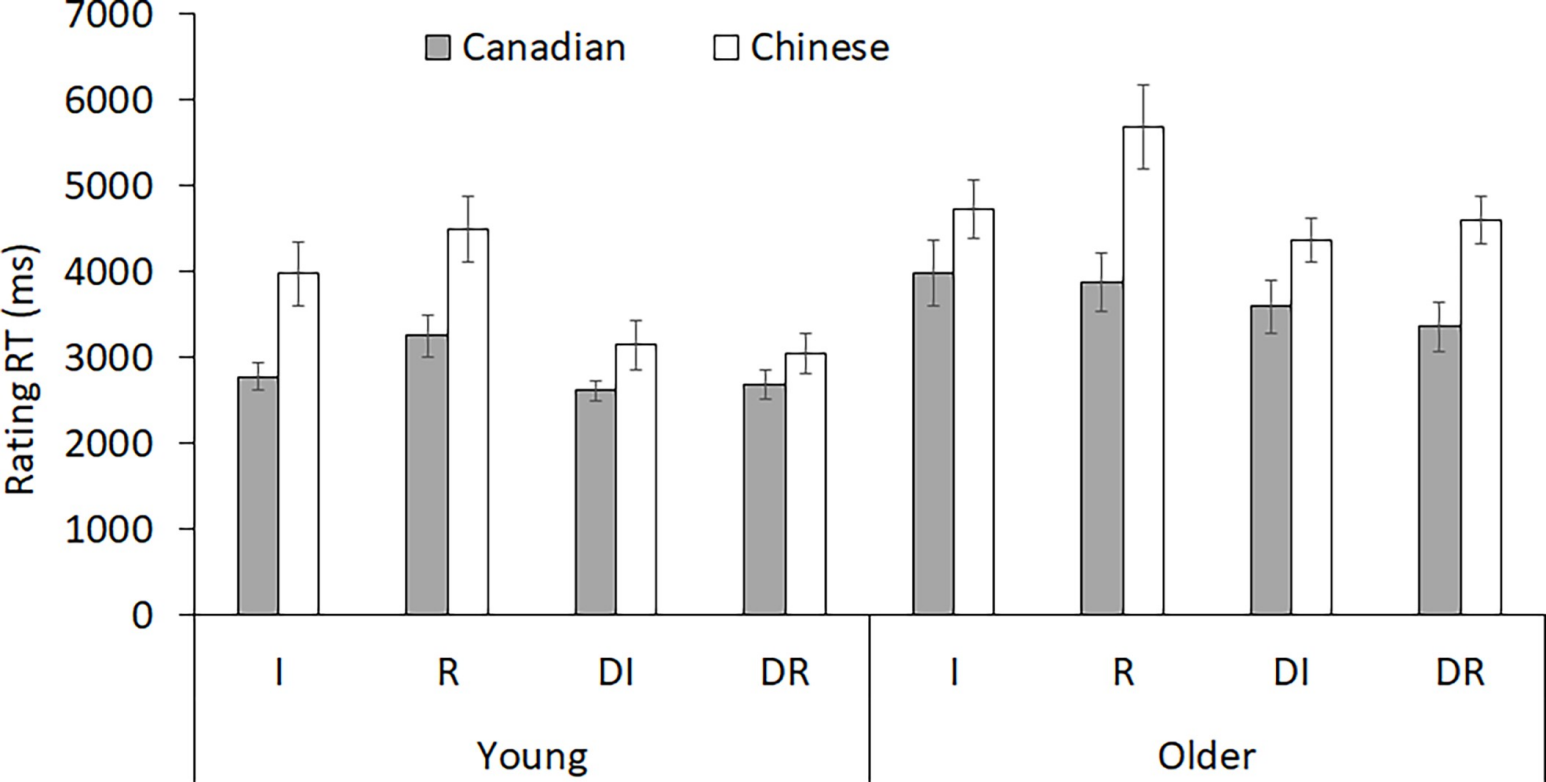
Rating RTs at encoding for each age and culture group across item types. R = Relational items; I = Independent items; DR = Daily life items in the “Relational” block, DI = Daily life items in the “Independent” block.

Fig 2 presents the raw rating RTs, after being trimmed by excluding outliers beyond 2.5 SDs away from the mean within each condition (R, I, D) for each participant, resulting an exclusion of 2.24% RTs. The ANOVA on log-transformed RT revealed a significant main effects of culture, *F*(1,139) = 18.07, *MSE* = 0.53, *p* < .001, η^2^ = 0.12, and age, *F*(1,139) = 18.64, *MSE* = 0.53, *p* < .001, η^2^ = 0.12. Rating was slower for Chinese (*M* = 4253.79, *SD* = 1950.92) and older (*M* = 4273.67, *SD* = 1910.95) than Canadian (*M* = 3273.09, *SD* = 1423.48) and young adults (*M* = 3252.93, *SD* = 1463.53) respectively. The main effect of item type was also significant, *F*(1.42, 196.73) = 22.40, *MSE* = 0.06, *p* < .001, η^2^ = 0.14. Pairwise comparisons with Bonferroni correction showed that daily life typicality ratings did not differ between the two blocks (DR: *M* = 3427.70, *SD* = 1625.03; DI: *M* = 3434.96, *SD* = 1659.27), *p* = 1.00; however, both tended to be rated faster than social context relevance ratings on I (*M* = 3871.54, *SD* = 2061.49) or R items (*M* = 4333.27, *SD* = 2417.81), *p*s < .01, with I items being rated marginally faster than R items, *p* = .06. This item type effect was qualified by an interaction with culture, *F*(1.42, 196.73) = 3.45, *MSE* = 0.06, *p* < .05, η^2^ = 0.02; and a marginally significant 3-way interaction, *F*(1.42, 196.73) = 3.24, *MSE* = 0.06, *p* = .06, η^2^ = 0.02. Follow-up analyses showed a larger effect size of the item type effect for Chinese (*F* = 38.96, η^2^ = .35) than Canadian participants (*F* = 3.20, η^2^ = .04). Specifically, the item type effect was present in all groups (*p*s < .01) except for Canadian older adults (*p* = .32). No other effects were significant (*p*s > .10).

To determine whether the cultural effects on encoding ratings and rating RTs could be attributed to other potential covariates, we conducted bivariate Pearson correlations between encoding performance and demographic variables (i.e., education, health rating, SCS independent, SCS interdependent, and SES-D) as displayed in Table 1. Only SCS interdependence score showed a marginally significant positive correlation with ratings at encoding (*r* = .16, *p* = .05). However, the results (particularly the cultural differences) remained the same after controlling for the SCS interdependence score as a covariate. No correlations were found significant between these variables and rating RT (*r*s < .14).

#### Self-reported context imagery vividness

The ANOVA on the context imagery vividness ratings (see Table 2) revealed significant main effects of all three factors: culture, *F*(1, 136) = 17.73, *MSE* = 1.12, *p* < .001, η^2^ = 0.11; age, *F*(1, 136) = 4.51, *MSE* = 1.12, *p* < .05, η^2^ = 0.03; and context, *F*(2, 278) = 12.44, *MSE* = 0.62, *p* < .001, η^2^ = 0.8. No interactions were significant (*p*s > .36). Specifically, the imagery vividness was rated higher by Chinese (*M* = 3.97, *SD* = 0.57) than Canadians (*M* = 3.54, *SD* = 0.66), and higher by young (*M* = 3.86, *SD* = 0.54) than older adults (*M* = 3.64, *SD* = 0.73), *p*s < .05. Additionally, pairwise comparisons with Bonferroni correction showed that imagery formed for experiential D context (*M* = 4.02, *SD* = 0.92) was rated more vivid than those for social I (*M* = 3.61, *SD* = 0.89) and R context (*M* = 3.63, *SD* = 0.94), *p*s < .001. The two social contexts (I and R) did not differ from each other (*p* = 1.00).

**Table 2:** Self-reported context imagery vividness during encoding.

	Canadian		Chinese	
Context	Young	Older	Young	Older
**D**	3.89 (1.05)	3.61 (0.96)	4.50 (0.77)	4.08 (0.60)
**I**	3.57 (0.85)	3.36 (0.96)	3.75 (0.87)	3.75 (0.84)
**R**	3.49 (0.85)	3.31 (1.09)	3.97 (0.77)	3.75 (0.91)

Each cell provides mean score, with standard deviation in parenthesis. D = Daily Life context; I = Independent context; R = Relational Context.

Furthermore, to evaluate whether higher rating and longer rating RT indeed reflected encoding quality (e.g., forming vivid imagery of context), we ran bivariate Pearson correlations between ratings, rating RTs and self-reported imagery vividness ratings. The results showed that context vividness was positively correlated with rating across all three contexts (*r* ranged from .20 to .31, *p*s < .05), but not with rating RT (*r* < .09, *p*s > .14).

### Encoding-memory relationship

To assess whether encoding was related to the variance in the subsequent memory performance, we conducted three sets of analyses: First, rating performance (rating and rating RT) was analyzed as a function of items’ context retrieval status (recognized vs. misrecognized); Second, correlation analyses were run between encoding and memory (item and context memory). Third, covariance analysis was conducted to examine whether the reported cultural effects on context memory would hold after controlling for rating performance. Covariance analysis was conducted only for context memory because cultural effect was found only in context but not in item memory [8].

#### Rating performance as a function of context recognition status

Ratings and log-transformed rating RTs were recoded according to each item’s context recognition status and then submitted to a 2 (culture) × 2 (age) × 2 (recognition status: recognized vs. misrecognized) ANOVA. The ANOVA on ratings replicated the culture (*p* < .001) and culture by age interaction (*p* < .05). Most interestingly, the main effect of context recognition status was significant, *F*(1,139) = 11.65, *MSE* = 0.09, *p* = .001, η^2^ = 0.08, with a higher rating for items with recognized context (*M* = 3.45, *SD* = 0.49) relative to those with misrecognized context (*M* = 3.33, *SD* = 0.53). This effect was further qualified by an interaction with age, *F*(1,139) = 8.61, *MSE* = 0.09, *p* < .05, η^2^ = 0.06. Recognition status effect being significant only for older (*p* < .001) but not for young adults (*p* = .75). No other effects were significant (*p*s > .19). The ANOVA on rating RTs replicated the main effects of age (*p* < .001) and culture (*p* = .001). No other effects were significant (*p*s > .12).

Driven by the hypothesis for cultural differences in encoding-memory relationship, we also ran separate univariate ANOVAs on rating and rating RT to examine context recognition status effect for each cultural group. The results showed the context recognition status effect was only significant for Chinese (*p* < .001 for rating, *p* < .05 for rating RT), but not for Canadian participants (*p* = .96 for rating, *p* = .19 for rating RT).

Taken together, objects with recognized context at retrieval were rated higher and slower at encoding relative to those with misrecognized context. This suggests a solid encoding-memory relationship, with higher and slower ratings associated with high-quality object-context encoding and thus better context recognition at retrieval. This relationship was more pronounced in older adults (in rating) and Chinese participants (in both rating and rating RT) than their age or culture counterparts respectively.

#### Rating-memory correlations

**Fig 3:**
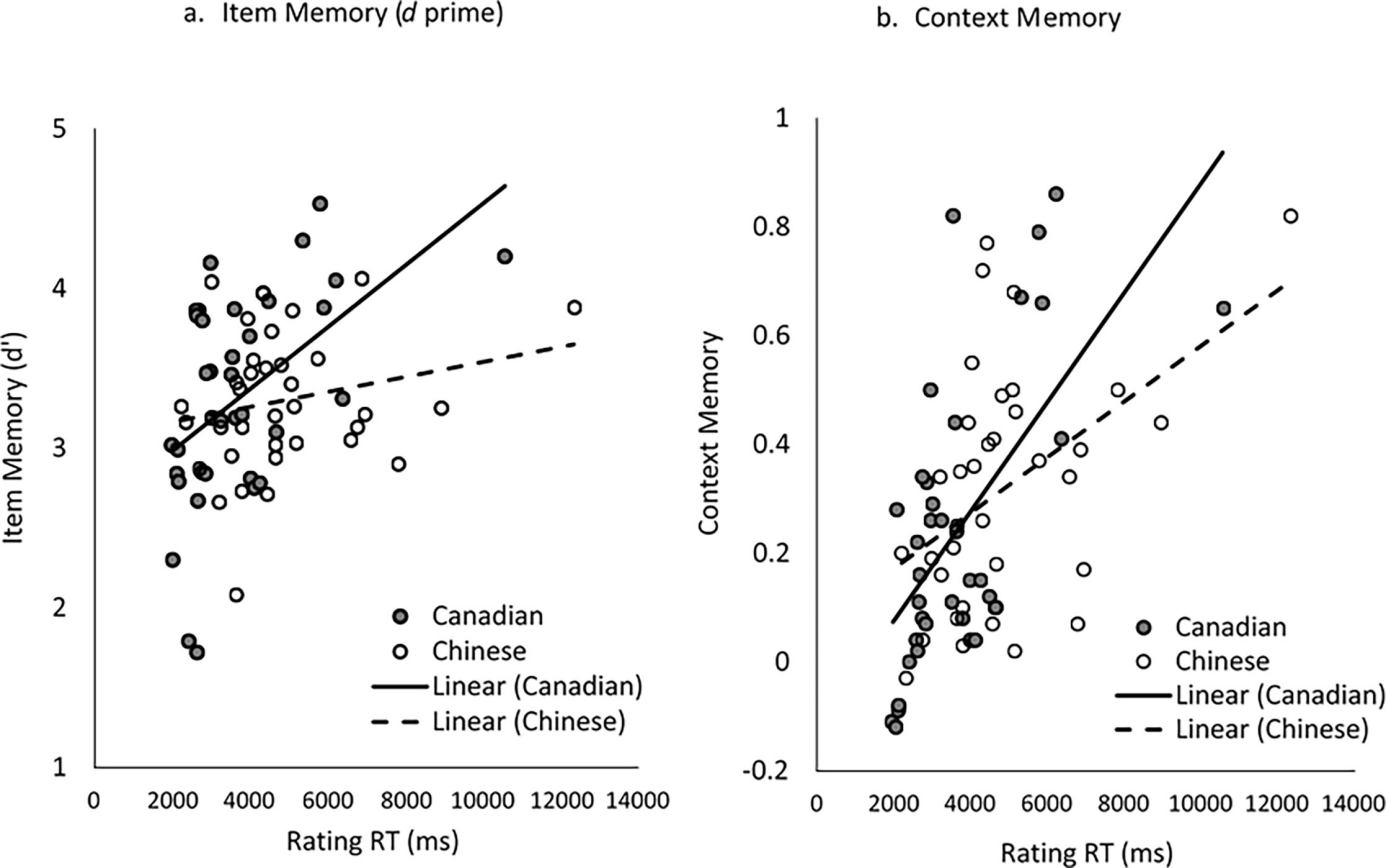
Scatter plots for the correlations between rating RT and memory in older adults. Panel a. item memory (*d* prime). Panel b. context memory.

Given the aforementioned age and cultural differences, we conducted bivariate Pearson correlations between rating and memory performance separately for each age and culture group (see Table 3). Overall, young adults in both cultures did not show any significant correlations, *r*s < .28, *p*s > .10. For older adults, Canadian participants showed positive correlations between rating RT and memory, across both item and context memory (*r*s > .50, *p*s < .01), whereas Chinese older adults’ positive correlation between rating RT and memory was exclusively demonstrated in context memory (*r* = .45, *p* < .01). Patterns remained the same when correlations were conducted with memory hits for both item and context memory. Fig 3 depicts the rating RT and memory correlations in older adults. Please note that rating-memory correlations were not significant across all groups, but these correlations may be spurious, considering the truncated range and limited variance of ratings (2.61–4.27, *SD* = 0.44).

**Table 3:** Correlations between rating and memory performance.

	Canadian		Chinese	
	Young	Older	Young	Older
	**Item Memory (*d’*)**			
**Rating**	.10	.21	.03	-.13
**Rating RT**	.05	.50**	.11	.21
	**Context Memory**			
**Rating**	.14	.29^	-.02	-.15
**Rating RT**	.25	.63**	.28	.45**

^*p* < .10

*p < .05

**p < .01.

#### Covariance analysis on context memory

To further determine the contribution of rating performance to the cultural effects on context memory, we included rating and rating RT (averaged across item types) as covariates to reanalyze context memory with age and culture as between-subjects variables, and block (I vs. R) as a within-subjects variable. Table 4 summarizes the *F* and *p* values for the critical culture and age effects for the ANOVA originally reported in Yang et al. [8] and the ANCOVA with rating and rating RT as covariates. Overall, compared to the original ANOVA [8], age effect remained significant (*p* < .001), but the cultural effect (*p* < .05) was no longer significant (*p* = .77) in the ANCOVA. The age by culture interaction was not significant in both analyses. Together with the correlation data, this suggests that the cultural differences in context memory reported in our previous work [8] might be largely accounted for by encoding differences between the two cultures.

**Table 4:** The Effects of age, culture, and age by culture interaction resulted from ANOVA [8] and ANCOVA.

	ANOVA^1^		ANCOVA^2^	
Effect	*F*	*p*	*F*	*p*
**Age**	104.82	.00	155.66	.00
**Culture**	5.28	.02	0.09	.77
**Age by Culture Interaction**	0.13	.72	0.21	.64

^**1**^The 2 (age) × 2 (culture) × 2 (block) ANOVA model, as reported in Yang et al. [8]

^**2**^The ANCOVA model including rating and rating RT as covariates.

## Discussion

The results of the current study revealed clear cultural differences in encoding of socially and/or experientially meaningful item-context associations. Chinese participants provided higher rating, took longer to rate, and reported more vivid imagery of encoding contexts than did their Canadian counterparts. Furthermore, the results suggest that the previously reported context memory advantage in Chinese may have its origin in the construction of elaborative and meaningful object-context associations at encoding.

### Cultural effects on encoding

At encoding, Chinese participants provided higher ratings, took longer to rate, and reported higher level of vividness in encoding context imagery than Canadian participants. The positive correlations between ratings and the self-rated vividness of the imagery suggest that the higher ratings of Chinese relative to Canadian participants may reflect their higher quality of object-context associations (e.g., enhanced vividness and/or more details) formed during encoding. Similarly, the longer rating time of Chinese participants may reflect their engagement of intentional elaborations in forming meaningful associations between items and their corresponding encoding contexts. This finding adds to literature on cultural differences in various cognitive processes, such as perception, attention and memory [19–21]. However, we note but were unclear of the explanations for the opposite directions of age differences in ratings between the two cultures. Nevertheless, cultural effect was preserved in older adults, as both age groups showed significant cultural effects. Taken together, the results suggest that Chinese participants tend to engage elaborative encoding to form better quality associations between objects and imagined social contexts, relative to their Canadian counterparts.

Of note, the results also revealed significant item type effects. Overall, D items tended to be rated higher and faster than the R/I items. This might be because that the typicality ratings in D context can be easily based on objective and perceptual information (e.g., frequency of personal experience with the object), whereas rating objects in the social I/R contexts require more semantic processing (e.g., mentally imagining a hypothetical social scenario and then forming meaningful associations between the object and this scenario). Consistent with this speculation, participants formed more vivid visual imageries for D context (*M* = 4.02, *SD* = 0.92) relative to I/R contexts (I context: *M* = 3.61, *SD* = 0.89; R context: *M* = 3.63, *SD* = 0.94), *p*s < .001. Furthermore, R items were rated lower than the other types of items, presumably due to the complexity of R context because it involves imagined inter-personal social interactions and relationship. Given their holistic processing tendency, Chinese participants might be more sensitive to the variance in contexts and thus showed more pronounced item type effect relative to their Canadian counterparts.

In addition, the cultural variations in rating performance were preserved into older age, as the cultural effects were shown in both young and older adults, despite of aging-associated general slowing in rating RT and decline in vividness of imageries formed for encoding contexts. The age-invariant cultural effect on encoding performance mimics the age-invariant cultural effect on context memory [8]. This is consistent with the theories on age differences in culture-cognition interaction, which proposed that the effect of cultural experience on cognitive processing tends to be maintained well into late adulthood [22–23].

Taken together, the encoding data suggest that Chinese participants, as compared to their Canadian counterparts, have a tendency to be more engaged in elaborative processing (i.e., longer rating time) when forming socially and/or personally meaningful object-context associations, and thus form more vivid and higher quality associations (i.e., higher ratings). They are also more sensitive to the context variance than Canadian participants at encoding. Nevertheless, these cultural variations tend to be preserved in older adults.

### Encoding-memory relationship

Relative to those objects with misrecognized contexts, objects with correct context recognition at retrieval were rated higher and slower at encoding. This strongly supports encoding-memory relationship (i.e., higher and slower ratings predicts the likelihood of correct context recognition), particularly for older and Chinese participants. We speculate that, the age differences might be due to age-related decline in cognitive resources [24]. Older adults’ context recognition may be more heavily contingent on the effort invested during encoding. Young adults, however, may be able to retrieve even those object-context pairs encoded with low effort. The cultural differences here, however, may reflect differences in context sensitivity. Chinese participants, driven by their holistic context processing inclination, may engage more elaborative context processing to form better quality object-context associations and are consequently more likely to use these associations to enhance context recognition.

Consistently, only older adults showed positive rating-memory correlations, further suggesting that context recognition of older adults is more determined by their encoding effort [25]. Although slower effortful encoding benefited older adults from both cultures on context memory, Canadian older adults also showed a benefit on item memory. The results suggest a processing inclination evenly distributed towards items (i.e., objects) and contexts for Canadians whereas there was a context-exclusive processing inclination in East Asians [3, 11]. As a consequence, Chinese older participants tend to invest time and effort to selectively and specifically focus on context processing which thus benefit their subsequent context memory. Following this reasoning, we speculate that slower encoding in Canadian older adults may be related to effortful processing of both individual objects and the object-context associations, but for Chinese older adults, slower encoding may be specifically related to the elaborative encoding of object-context associations.

Furthermore, the covariance analysis showed that the age effect on context memory preserved whereas the cultural effect disappeared after controlling for rating performance. This suggests that the cultural variations, but not age differences, in context memory may be driven by the encoding differences. In line with Baltes [22], we proposed that socially and/or personally meaningful item-context binding may recruit culturally-relevant knowledge, experiences, and/or strategies, and thus allows Chinese participants to adopt culturally-favored holistic context-sensitive processing at encoding, which thus benefits their subsequent context memory performance. However, age-related context memory deficits and general slowing in encoding may be primarily driven by biological and neurological aging process, with little relevance to the cultural variation in processing style [9, 22].

### Limitations

Nevertheless, this study also has limitations as noted below. First, cultural differences in encoding and memory might be explained by alternative factors. For instance, it could be that Chinese participants are generally more compliant with the task instruction and/or are more motivated to perform well than their Canadian counterparts. If this were the case, we would expect cultural differences across all different tasks. However, we did not find cultural differences in item memory [8]. In addition, other studies did not report cultural effects in context/source memory [26–27]. For example, Chua and colleagues reported no cultural differences in source memory for four speakers who introduced different statements during the study phase [26]. Yang and colleagues reported a source memory advantage in Canadian relative to Chinese participants for information processed categorically, a style generally favored by Western culture [27]. Based on these inconsistencies, we argued that the task-general motivation/compliance may not play an important role here. However, we should admit that there might be task-specific motivation/compliance effect. Presumably, the culturally preferred encoding/processing tasks might capture more attention and better promote task engagement.

The second limitation is with respect to the assumption that a longer time to generate ratings reflects more engagement of elaborate encoding processes. This seemingly counters to the classical empirical work by Craik and Tulving [28], which revealed equivalent processing time between deep and shallow encoding strategies. In the current study, however, only one encoding strategy (i.e., rating according to a social scenario) was engaged. It is possible that individual differences in encoding time indeed reflect how well they engage in this specific strategy. Similarly, when the effect of different strategies was examined within-participants (Experiment 1) [28], longer response time was consistently associated with deeper processing and better memory.

To conclude, the current study provides the first empirical evidence for cultural effects on encoding objects in imagined meaningful social or experiential contexts and this encoding difference may contribute to the cultural effect on subsequent context memory performance [8].

## Supporting information

S1 File The final SPSS dataset “S1_file_PONE-D-18-05261_final data set”. (SAV)
